# Agreement Between a Pre-Markered T-Shirt and Manual Marker Placement for Opto-Electronic Plethysmography (OEP) Measures

**DOI:** 10.3390/s25144464

**Published:** 2025-07-17

**Authors:** Nayani G. Adhikari, Eugénie Hunsicker, Matthew T. G. Pain, John W. Dickinson, Samantha L. Winter

**Affiliations:** 1 School of Sport, Exercise and Health Sciences, Loughborough University, Loughborough LE11 3TU, UK; nadhikar@sgul.ac.uk (N.G.A.); m.t.g.pain@lboro.ac.uk (M.T.G.P.); 2 Population Health Research Institute, City St George’s, University of London, London SW17 0RE, UK; 3 Department of Mathematical Sciences, Loughborough University, Loughborough LE11 3TU, UK; e.hunsicker@lboro.ac.uk; 4 School of Sport and Exercise Sciences, University of Kent, Canterbury CT2 7NZ, UK; j.w.dickinson@kent.ac.uk

**Keywords:** breathing pattern disorder, motion capture technique, opto-electronic plethysmography, phase angle, tidal volume

## Abstract

Opto-electronic plethysmography (OEP) is used to measure chest wall compartment volumes and their synchronisation. Breathing pattern disorder (BPD) can be distinguished using the phase angles between these chest wall compartments during exercise. However, the time taken to manually place the standard OEP model involving 89 reflective markers is high during clinical application. The purpose of this study was to investigate the use of a pre-markered T-shirt instead of markers applied directly to the skin at rest, during different exercise intensities and recovery. Thirty-nine healthy participants (24 male, 15 female) aged 18–40 years performed an incremental cycling test with the skin-mounted OEP marker set. Participants then repeated the same cycling test with a pre-markered T-shirt. Across all test conditions, the T-shirt showed a strong level of agreement (Intraclass correlation coefficient (ICC) ≥ 0.9) with the standard breath-by-breath (BbB) gas analyser. Moreover, ICC values exceeded 0.8 for compartment contributions across all test conditions, indicating excellent agreement with the skin-mounted markers. The phase angles between compartments showed the best agreement during the moderate exercise level (0.6 < ICC < 0.8). In conclusion, the pre-markered T-shirt presents a viable solution for the quick monitoring of breathing patterns, as well as an effective tool in diagnosing BPD during exercise.

## 1. Introduction

**Figure 1:**
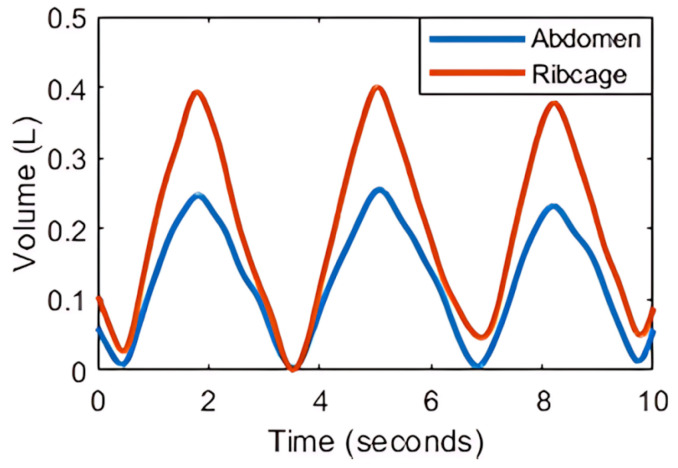
Volumes of chest wall compartments, including abdomen (AB) and ribcage (RC), determined by opto-electronic plethysmography (OEP), showing changes in compartment volume over time.

Opto-electronic plethysmography is a 3D motion capture technique that tracks chest wall movements using three-dimensional coordinates of retro-reflective markers placed on the torso [1]. This technique assesses chest wall compartment contributions, including pulmonary ribcage (RCp), abdominal ribcage (RCa) and abdomen (AB), and also breathing patterns at rest and during exercise (Figure 1). Therefore, OEP is used in respiratory monitoring in healthy and various pathological groups [2].

Typically, OEP requires the placement of 89 markers on the torso [1,3]. A full set of 89 OEP markers is used in many studies due to its capacity to monitor a wider range of breathing parameters, including thoracoabdominal asynchrony [4]. This is a process that is both time-consuming and labour-intensive in a clinical setting. Practically, it takes a trained operator at least 20 min just to perform marker placement. Furthermore, affixing markers to double-sided adhesive tapes requires significant staff time. This task needs to be performed before the patient arrives, and the markers also need to be removed afterwards. Moreover, placing markers directly on the skin can be inconvenient and uncomfortable for patients, and removing them can be equally awkward. Additionally, some participants may have allergies to adhesive tape, and the presence of body hair complicates the process.

Wearable motion sensors are useful in taking physiological measurements during exercise [5,6]. The advantage of the OEP technique arises from the ability to compute compartment volumes and phase angles during exercise [7,8]. While simpler wearable systems have been proposed, these are usually limited to the measurement of the whole breath tidal volume [9]. OEP has been used to validate some more complex wearable systems, but only at rest [10,11,12]. To date, no prior work has established a validation of the phase angle and percentage contributions during exercise, which are key breathing metrics that can be derived from the OEP system and which are important for both clinical and sporting applications.

A pre-markered T-shirt significantly reduces the preparation time for participants and staff while preserving the utility of the OEP procedure. It is reusable, further minimising the time required for both parties. While the pre-markered T-shirt addresses the above issues, it introduces a potential drawback, which is the lack of customisation. Each patient is unique, and placing markers directly on the skin can be tailored to individual anatomical variation. Therefore, the key question is whether the time savings and increased comfort provided by the pre-markered T-shirt justify the possible loss of individualisation. The current study involves a body-fitting T-shirt with an OEP marker set pre-attached to the T-shirt to monitor participants’ breathing patterns not only at rest but also during exercise.

There is a significant lack of studies based on OEP in female participants during both static and dynamic tasks. Additionally, most studies related to OEP are limited to determining a few breathing parameters, including changes in the chest wall compartmental volume, breathing frequency, inspiratory time, expiratory phases, and tidal volumes [12].

One potential clinical application of OEP is diagnosing breathing pattern disorder (BPD). BPD leads to respiratory issues that affect quality of life. It refers to a group of respiratory conditions due to irregular breathing patterns that require more involved metrics for diagnosis. Thoracoabdominal asynchrony is one such type of BPD, which introduces a lack of coordination between the thoracic and abdominal regions [7]. Phase angle is a measure of the temporal movement of one torso compartment in relation to another during each breath and is commonly used to estimate thoracoabdominal asynchrony [4,13]. OEP is uniquely useful in identifying chest sub-compartment contributions and synchrony between compartments during exercise. For example, BPD can be distinguished using the phase angles between RC and AB, RCp and RCa, RCa and shoulder, and RCp and shoulder during exercise [8]. Therefore, if the pre-markered T-shirt can be used to monitor these important breathing parameters for diagnosing BPD, it can be considered as a valuable respiratory monitoring tool.

Hence, for the first time, this study evaluated the agreement of a pre-markered T-shirt over a skin-mounted OEP marker set, considering a wide range of breathing parameters, especially compartment contributions and phase angle parameters, which help diagnose BPD. This study validated the pre-markered T-shirt over a standard breath-by-breath (BbB) gas analyser used at the same time and a skin-mounted OEP marker set on the same day, at rest, during incremental cycling exercise, and at recovery. Moreover, the study checked the reliability of the pre-markered T-shirt between days at rest and also used a considerably large sample, including both male and female healthy participants.

Participants performed the experiment either wearing a pre-markered T-shirt or with markers mounted directly on the skin on a bare chest for men and over a sports bra for women. These two conditions will be referred to as T-shirt and skin-mounted in the manuscript.

## 2. Materials and Methods

### 2.1. Participants

Thirty-nine adult participants (18–40 years old, male and female) who are physically safe to complete exercise tests on a cycle ergometer participated in the study. Before testing, participants were asked to fill out a consent form and a health screen questionnaire, which were approved by the Loughborough University Ethics Committee (Approval number: LEON 8294). However, excessive marker drop-out files were excluded from the final analysis. The sample size for the final analysis and a summary of participants’ demographic and anthropometric data are given in Table 1 for each test condition, including rest, low, moderate, and high-intensity exercise, and recovery.

### 2.2. Equipment

A BbB gas analyser (Cortex Biophysik Metalyzer 3B, CORTEX Biophysik GmbH, Leipzig, Germany) was used simultaneously with the motion capture system consisting of 10 Qualisys cameras (Miqus M5, Qualisys AB, Goteborg, Sweden). Prior to each participant’s testing session, the camera system calibration was performed using an L-frame and a wand (wand kit 300 mm-carbon fibre, Qualisys AB, Goteborg, Sweden) at a sampling rate of 100 Hz. OEP data were also recorded at 100 Hz.

**Figure 2:**
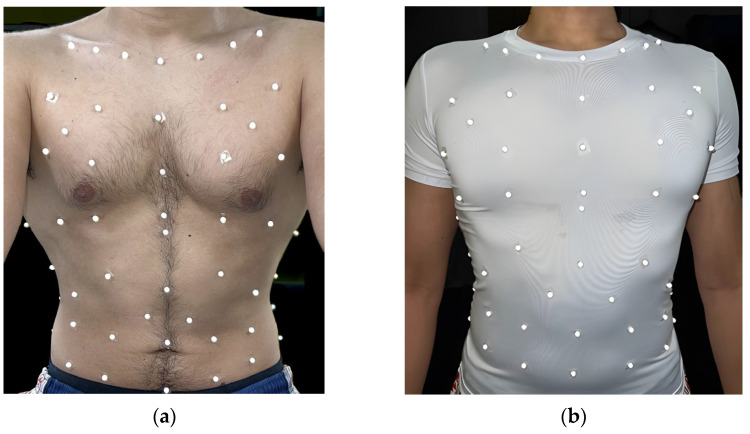
(**a**) Skin-mounted marker placement; (**b**) The pre-markered T-shirt (Polyester/Elastane) was based on 89 reflective markers (6.4 mm diameter) pre-attached to the T-shirt to monitor breathing using opto-electronic plethysmography (OEP).

A cycle ergometer (Lode Corival Eccentric, Groningen, Netherlands) was used to perform a loaded cycling exercise protocol. Additionally, a pre-markered T-shirt (95% Polyester and 5% Elastane) was used as shown in Figure 2. Eighty-nine small markers were placed on the stretchy slim-fit T-shirt according to a grid-like pattern [3]. This marker placement was permanently markered on the T-shirt to be consistent. Markers were placed before the participant wore the T-shirt (pre-markered) by a trained operator. An extra C7 (cervical vertebra) marker was placed separately on the skin after wearing the T-shirt as a reference point [10].

The suitable T-shirt size was selected depending on their gender and size. The best-fit size was chosen according to the participant’s body structure. The T-shirt was made of slightly stretchable fabric, which did not alter the participant’s natural breathing pattern due to being overly tight.

### 2.3. Protocol

The pre-markered T-shirt was used on two testing days using two protocols. These two protocols were assigned randomly. In the first protocol, the participant was asked to put on the T-shirt, and chest wall motions were recorded using the motion capture system while the participant was seated on the exercise bike during the resting phase with normal breathing for a minute. No exercise was performed with the T-shirt in protocol one.

In the second protocol, participants performed two exercise tests: one with the skin-mounted OEP marker set and one with the T-shirt. A trained operator placed markers on the participant’s torso, and the participant performed an incremental cycling exercise test on the cycle ergometer. Data was recorded in five conditions, including rest, low, moderate, and high-intensity exercise, and recovery. After the rest phase with one minute of normal breathing, participants performed the incremental cycling test starting at 50 W/70 W, depending on their physical activities taken from the health questionnaire. Most participants were set at 50 W, but if their reported physical activities were extremely high, the starting load was set at 70 W. Participants were asked to maintain a pedalling frequency of 70 ± 5 revolutions per minute (rpm). The load was then incremented until the participant achieved the required respiratory exchange ratio (RER)/ratings of perceived exertion (RPE) values at that level according to the Borg scale [14]. The exercise intensities were defined based on the RER obtained from the BbB gas analyser and RPE values, classifying low, moderate, and high intensities as RER values of 0.8, 1.0, and >1.1 and RPE values of 11, 13/14, and 17/18, respectively [14,15]. After 3 min of cycling at each exercise intensity and recovery, OEP data were collected for a maximum of 60 s.

After a rest of at least 20 min or when the participant felt able to start again, the cycling test was repeated wearing the T-shirt at rest, during all exercise intensities and recovery, as explained above, using similar loads for the increment. In protocol two, the BbB gas analyser was simultaneously used to monitor respiratory functions in real time.

### 2.4. Data Analysis

The BbB gas analyser data were recorded and exported using the CORTEX Biophysik MetaSoft CPX software (CORTEX Biophysik GmbH, Leipzig, Germany). The BbB gas analyser has a restricted capacity to obtain a limited set of breathing parameters, especially since it does not include compartment volumes and many other breathing parameters that can be derived using OEP. However, the BbB gas analyser is the best available method for validating large-scale respiratory measurements. Therefore, this comparison focused only on three timing and ratio parameters: tidal volume (Vt), respiratory rate (RR), and inspiratory time (tI). Breath-by-breath data of three breathing parameters obtained simultaneously from both systems were synchronised by aligning the timing indices of each trial. Then, these breath-by-breath data were averaged for each trial to compare averages between the two systems.

The OEP data obtained with both the skin-mounted marker set and the pre-markered T-shirt were processed similarly using Qualisys Track Manager (QTM) (Version 2023.2) and gap-filled up to 20 frames. A 60 s segment was first extracted from the end of each test condition during steady-state breathing [16]. If larger gaps (>20 frames) were present, the segment was trimmed accordingly to retain a portion representing approximately 8–10 complete breaths with all markers visible. Then, the QTM file was exported to MATLAB (Mathworks Inc., Natick, MA, USA). Custom-written MATLAB (Version R2020b) scripts were used to filter marker data and derive breathing parameters. The original marker data were filtered using a zero-lag, low-pass fourth-order Butterworth filter with a cutoff frequency of 2 Hz. The torso and compartment volumes over time were calculated using a prism-based method [3].

Breathing parameters included timing and ratio, compartment contributions, and phase angle parameters. RR, Vt, tI, minute ventilation (VE), expiratory time (tE), total breath time (tTot), time taken to reach peak expiratory flow (tPTEF) and time taken to reach peak inspiratory flow (tPTIF) were considered as timing and ratio parameters. The percentage compartment contributions included RCp contribution (RCpCT), RCa contribution (RCaCT), RC contribution (RcCT), AB contribution (AbCT), combined RCa and AB contribution (RCaAbCT), and the ratio of RC volume to AB volume (RcAbIndex). Phase angles were calculated between RC and AB (RcAbPhase), between RCp and AB (RCpAbPhase), between RCp and RCa (RCpRCaPhase), between RCa and shoulder (RCaSPhase), between RCp and shoulder (RCpSPhase) and between AB and shoulder (AbSPhase).

### 2.5. Statistical Analysis

Vt, RR, and tI measured from the BbB gas analyser and the OEP system using the T-shirt were compared. These comparisons were conducted using Bland–Altman analysis [17] and intraclass correlation coefficients (ICCs). The agreement was then evaluated in terms of the ICC values of breathing parameters obtained by the OEP system with the pre-markered T-shirt and markers directly placed on the bare chest on the same day in protocol two. In addition, ICC values were calculated to check the reliability of the T-shirt over two days, only at rest. To calculate ICC values, a two-way mixed model was used, considering the absolute agreement with the average measures [18]. The significance of the ICC values was determined using an F-test [19].

## 3. Results

### 3.1. Comparison of the Pre-Markered T-Shirt with the BbB Gas Analyser

Both systems displayed a strong level of agreement, with ICC values greater than or equal to 0.9 in all conditions for Vt, RR, and tI (Table 2). However, the 95% CIs of the ICC values were slightly wider at rest for Vt and across all levels, except for high intensity for RR. This indicates a higher variability in the data, particularly for RR, which will be highlighted in the discussion. The inspiratory time, tI, consistently showed excellent agreement with the two systems, with ICC values exceeding 0.9 and narrow 95% CIs across all levels.

The average values of bias and percentage bias are reported for three breathing parameters in each condition (Table 2). The highest mean bias value of 0.13 ± 0.13 (L) for Vt was found at recovery, while the lowest was 0.07 ± 0.08 (L) at rest. The highest mean bias value of 2.12 ± 1.53 breaths per minute for RR was found during the moderate exercise level, while the lowest was 1.23 ± 0.97 breaths per minute at rest. The highest mean bias value of −0.064 ± 0.117 (s) for tI was found at recovery, while the lowest was 0.005 ± 0.079 (s) during the high level of exercise. The tI parameter consistently showed a very low bias for both males and females across all exercise levels. The highest percentage bias was shown in Vt compared to the other two variables. Noticeably, the lowest average percentage bias was reported during high-intensity exercise for all parameters (Vt: 7.93%, RR: 5.77%, and tI: 0.74%). The maximum bias was reported in recovery for Vt (11.97%) and tI (−5.94%) and the second highest was reported for RR (9.67%). Generally, the above results showed that the T-shirt has good agreement with the BbB gas analyser, which is currently used as a standard method in respiratory assessments.

**Figure 3:**
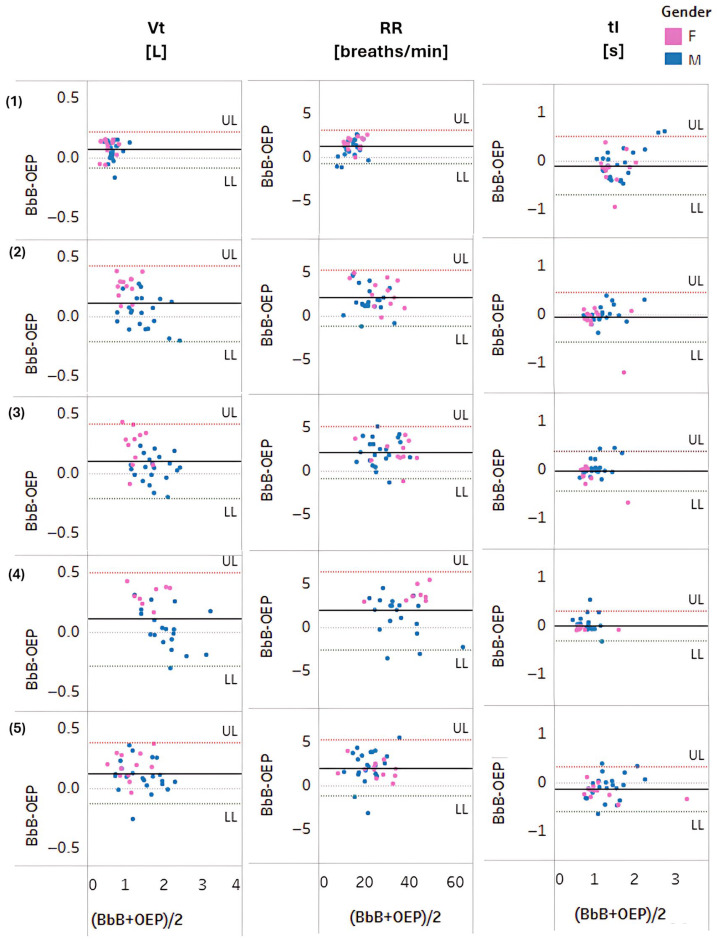
Bland–Altman plots for Vt, RR and tI at (**1**) rest, during (**2**) low, (**3**) moderate, and (**4**) high-intensity exercises, and at (**5**) recovery over male (blue) and female (pink) participants. The black thick line shows the average difference (bias), whereas the dotted lines show the 95% upper and lower limits of agreement (LOA); Upper Limit (UL) = average difference + 1.96 × SD of the difference, Lower Limit (LL) = average difference − 1.96 * SD of the difference.

Bland–Altman plots revealed that greatest differences between the two systems were within the 95% limits of agreement, without a distinct trend suggesting good overall agreement (Figure 3). However, there was a female outlier for tI at rest, low and moderate intensity, with comparatively higher tI values. These outliers were due to two highly trained female athletes (>10 h of physical activity per week) and were associated with breath-holding at the end of the inhale that was visually observed during data collection, and which was reflected in the OEP measure but not reflected in the BbB gas analyser data.

### 3.2. Comparison of the Pre-Markered T-Shirt with Skin-Mounted OEP Marker Placement

**Figure 4:**
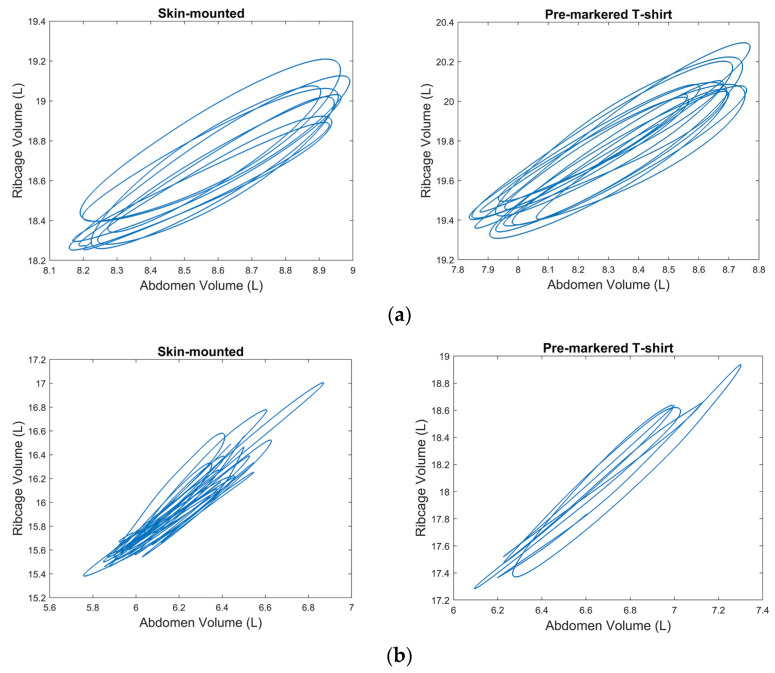
Volume traces for the phase angle between ribcage (RC) and abdomen (AB), obtained from the skin-mounted marker system (left) and the pre-markered T-shirt system (right). Data are shown for (**a**) participant no. 22 (male) and (**b**) participant no. 27 (female) during low-intensity exercise.

Figure 4 presents representative traces for the phase angles between RC and AB obtained from the skin-mounted marker and the pre-markered T-shirt trials. Although the trials were not conducted simultaneously, the results still indicate similar breathing patterns for the two methods. Small discrepancies may be attributed to minor shifts in fabric alignment, marker positioning, as well as natural between-trial variability in the breathing pattern across trials.

Table 3 displays the ICC values calculated to evaluate the agreement of the T-shirt with the skin-mounted marker placement. Most ICC values calculated for breathing parameters across all conditions were statistically significant (*p* ≤ 0.05), with a few exceptions. These exceptions primarily involved timing ratio parameters, including tI/tE, tI/tTot, and RCpAbPhase. The ICC values were greater than 0.8 across all conditions for compartment contribution parameters, indicating excellent agreement with the skin-mounted OEP marker placement [20]. RCaCT had slightly lower ICC values compared to RCpCT and AbCT, as it is smaller than the other two compartments.

All ICC values for the phase angle parameters were significant at rest and during exercise. However, RcAbPhase, RCpAbPhase, and AbSPhase had lower ICC values at around 0.4 during recovery compared to the other phase angles. These three phase angles are related to the AB compartment, which has a higher impact when people slump during the recovery phase. Therefore, the pre-markered T-shirt is not ideal for use at the recovery stage when aiming to monitor these phase angle parameters. Across all conditions, a moderate exercise intensity showed the best agreement (ICC = 0.6 to 0.8) with the T-shirt and skin-mounted marker placement for phase angle parameters. The parameters measured during high-intensity exercise demonstrated the highest agreement, possibly because participants’ breath-to-breath variation reduces at this stage.

In addition, the day-to-day reliability of the T-shirt for compartmental contribution parameters was significant and high (ICC > 0.9), indicating the consistent marker positioning of the T-shirt over days at rest. However, the timing and ratio parameters and phase angles showed ICC values ranging from 0.4 to 0.7, with borderline significance for some parameters. However, tI/tE, tI/tTot, tPTEF, and AbSPhase did not have good day-to-day reliability, which may be associated with variations in breathing between days.

## 4. Discussion

For the first time, this study established the agreement between a pre-markered T-shirt and the bespoke placement of 89 skin-mounted OEP markers to monitor the breathing patterns of healthy individuals at rest, during exercise, and at recovery as a time-efficient alternative to traditional OEP marker placement. This evaluation included a wide range of breathing parameters that help identify thoracoabdominal asynchrony [8]. Moreover, the study evaluated the agreement between the T-shirt with the standard BbB gas analyser. The agreement with the BbB gas analyser was very good; in particular, tI was excellent during exercise. The compartment contributions measured from the T-shirt had excellent agreement with the skin-mounted placement, and the day-to-day reliability was excellent for compartment contributions at rest. Therefore, the pre-markered T-shirt did compare well with the skin-mounted placement for these OEP parameters.

The respiratory rate (RR) was more biased between the T-shirt and the BbB gas analyser due to differences in the calculation methods used between the two systems. The RR of the OEP system was calculated by dividing the number of breaths in the OEP trial by the trial length in minutes. The RR of the BbB gas analyser was calculated by taking the average of the breathing frequency given for each breath over the considered period of the OEP trial. The BbB gas analysis output reports a predicted respiratory rate alongside each breath based on the time of that breath. However, it is important to note that RR is not typically a primary parameter derived from OEP, as it is easily measured by a variety of simple techniques. Instead, the main OEP parameters of interest in this study were the percentage contributions and phase angles of chest wall compartments. Therefore, while some bias in RR is expected due to methodological differences, it does not substantially affect the validity of this study’s main findings.

Supporting previous findings, the RCa compartment showed the lowest agreement compared to the RCp and AB compartments due to its smaller size [21]. Important phase angles for distinguishing disordered breathers, including RcAbPhase, RCpRCaPhase, RCaSPhase, and RCpSPhase, had significant ICC values across all conditions, with the best performance during moderate-intensity exercise [8]. The between-day reliability of the T-shirt at rest demonstrated moderate reliability for most breathing parameters, except for the compartment contribution parameters. However, the compartment contribution measurements showed excellent consistency (ICC > 0.9), aligning with previous findings comparing the T-shirt to skin-mounted OEP placement. On the other hand, the timing parameters, including tI/tE, tI/tTot, and tPTEF, were unreliable between days, supporting earlier findings regarding the skin-mounted OEP marker set.

A previous study looked at the agreement between an OEP system and a smart textile and showed a Vt bias of 0.09 ± 0.15 L between the systems [12]. The current study demonstrated an even lower bias of 0.07 ± 0.08 L for Vt between the T-shirt and the BbB gas analyser at rest.

The present study had some practical limitations. A few trials had to be excluded from the study due to marker dropouts, especially during the high-intensity exercise. That led to different sample sizes for the five test conditions. This was associated with the participant slumping while cycling on the ergometer. Moreover, these body movements sometimes resulted in the T-shirt rolling up, leading to marker dropouts. The error in assessing tidal volumes through wearables increases when the subjects are involved in dynamic tasks [9].

**Figure 5:**
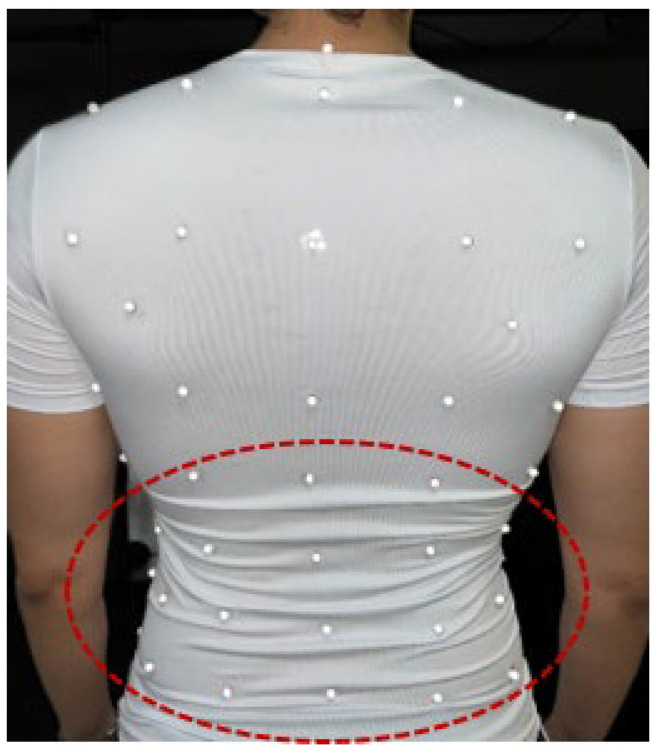
Posterior view of the pre-markered T-shirt. The red circle shows the wrinkles that appeared due to poor fit.

A suitable T-shirt size was chosen if the pre-markered T-shirt appeared to be properly fitted to the participant’s body. However, with the female participants, the actual placement shifted up and down underneath the breast marker row, even with different sizes of female T-shirt. Although the T-shirt size matched, these markers tended to be missed when capturing data, especially during cycling exercises. Therefore, the sample size at high-intensity exercise was considerably lower than at other levels. In addition, the variety in body shapes made it challenging to achieve an optimal fit with the existing T-shirt design [10]. Even if it fitted perfectly on the front, sometimes it did not perfectly fit the bottom half of the back, causing wrinkles (Figure 5).

These are important points to take into account in future studies when aiming to improve the pre-markered T-shirt. The current T-shirt, made from a stretchable fabric comprising Polyester and Elastane, was selected for its compression qualities. This material choice aimed to ensure that it would not excessively compress and thereby disrupt natural breathing patterns. However, considering the issue mentioned above, using pre-made body stocking material for the T-shirt design would be preferable for a personalised fit in future improvements. Additionally, for females, slightly sculpted shapewear tops could help address under-bust area issues and reduce wrinkling and flaring out from the lower back to the hips.

## 5. Conclusions

In conclusion, the pre-markered T-shirt is distinguished by its simplicity, time efficiency, and non-invasive methodology for monitoring breathing patterns. It showed excellent alignment with the BbB gas analyser. In addition, it demonstrated promising agreement with the skin-mounted OEP marker placement for most of the breathing parameters considered in this study, especially for compartment contributions. This suggests that the T-shirt is feasible for tracking chest wall movements at rest, during exercise, and at recovery. Therefore, the T-shirt serves as an effective screening tool, particularly for individuals with a low BMI, such as sports teams, enabling the quick assessment of breathing patterns. However, tracking certain breathing parameters, especially phase angle parameters, during recovery using the T-shirt is not optimal. The inconsistent breathing patterns, such as sighs, irregular rhythms, and shallow breathing, increase the bias and reduce the reliability at the recovery phase. The main findings drawn from this study suggest that the pre-markered T-shirt presents a viable solution; however, it needs further improvements, particularly concerning the fabric.

## Figures and Tables

**Table 1:** Demographic and anthropometric data (Mean ± SD) of participants and final sample size of each test condition.

Test Condition	Sample Size	Age (Years)	Height (m)	Body Mass (kg)	BMI (kg/m^2^)
Rest	M (n = 22)	25.2 ± 3.8	1.76 ± 0.07	77.37 ± 6.93	24.91 ± 2.54
	F (n = 13)	24.5 ± 4.1	1.67 ± 0.06	64.14 ± 7.22	23.08 ± 2.02
Low	M (n = 21)	25.1 ± 3.9	1.76 ± 0.07	76.91 ± 6.75	24.86 ± 2.59
	F (n = 12)	24.3 ± 4.2	1.67 ± 0.06	64.48 ± 7.43	23.08 ± 2.11
Moderate	M (n = 21)	25.1 ± 3.9	1.76 ± 0.07	76.91 ± 6.75	24.86 ± 2.59
	F (n = 11)	24.3 ± 3.9	1.68 ± 0.07	63.98 ± 7.57	22.82 ± 1.99
High	M (n = 19)	25.5 ± 3.8	1.76 ± 0.07	77.32 ± 6.58	24.91 ± 2.54
	F (n = 08)	23.6 ± 3.4	1.68 ± 0.07	64.48 ± 7.43	23.08 ± 2.11
Recovery	M (n = 22)	25.2 ± 3.8	1.77 ± 0.07	77.37 ± 6.93	24.91 ± 2.54
	F (n = 12)	24.3 ± 4.2	1.67 ± 0.06	64.48 ± 7.43	23.08 ± 2.11

**Table 2:** Mean ± SD of Vt, RR, and tI obtained from the OEP system with the pre-markered T-shirt and BbB gas analyser, mean ± SD of bias and percentage bias, intraclass correlation coefficients (ICCs) and their 95% CIs comparing both systems.

Test Condition	BbB Analyser	Pre-Markered T-Shirt	Bias	Percentage Bias (%)	ICC (95% CI)
Tidal Volume (Vt) (Litres)					
Rest	0.67 ± 0.17	0.60 ± 0.16	0.07 ± 0.08	11.62 ± 14.31	0.90 (0.51, 0.96)
Low	1.36 ± 0.39	1.24 ± 0.47	0.12 ± 0.16	11.38 ± 14.87	0.95 (0.81, 0.98)
Moderate	1.62 ± 0.38	1.52 ± 0.44	0.10 ± 0.16	8.31 ± 13.00	0.95 (0.83, 0.98)
High	1.97 ± 0.48	1.86 ± 0.58	0.11 ± 0.20	7.93 ± 12.70	0.95 (0.87, 0.98)
Recovery	1.37 ± 0.45	1.24 ± 0.47	0.13 ± 0.13	11.97 ± 12.93	0.96 (0.71, 0.99)
Respiratory Rate (RR) (breaths per minute)					
Rest	15.66 ± 3.72	14.42 ± 3.33	1.24 ± 0.97	7.79 ± 7.04	0.95 (0.41, 0.99)
Low	25.12 ± 6.61	23.03 ± 6.88	2.09 ± 1.62	9.79 ± 9.51	0.96 (0.48, 0.99)
Moderate	30.62 ± 7.47	28.50 ± 7.52	2.12 ± 1.53	7.77 ± 6.19	0.97 (0.47, 0.99)
High	38.43 ± 9.65	36.46 ± 9.92	1.97 ± 2.29	5.77 ± 6.46	0.98 (0.86, 0.99)
Recovery	24.04 ± 6.63	22.00 ± 6.51	2.04 ± 1.64	9.67 ± 8.58	0.96 (0.50, 0.99)
Inspiratory Time (tI) (seconds)					
Rest	1.56 ± 0.43	1.61 ± 0.36	−0.05 ± 0.16	−3.88 ± 9.27	0.96 (0.92, 0.98)
Low	1.17 ± 0.37	1.19 ± 0.37	−0.02 ± 0.13	−2.11 ± 8.74	0.97 (0.94, 0.98)
Moderate	0.99 ± 0.30	1.00 ± 0.30	−0.01 ± 0.10	−1.28 ± 8.66	0.97 (0.94, 0.99)
High	0.82 ± 0.24	0.82 ± 0.25	0.00 ± 0.08	0.74 ± 8.82	0.98 (0.95, 0.99)
Recovery	1.26 ± 0.51	1.32 ± 0.51	−0.06 ± 0.12	−5.94 ± 9.80	0.98 (0.95, 0.99)

**Table 3:** Intraclass correlation coefficients (ICCs) of breathing parameters at rest, during exercise, and at recovery, evaluating the agreement between the skin-mounted OEP system and pre-markered T-shirt and the day-to-day reliability of the pre-markered T-shirt at rest.

Breathing Parameter	Test Condition					Day-to-Day Reliability
	Rest	Low	Moderate	High	Recovery	Rest Only
Timing and Ratio Parameters						
RR	0.71	0.85	0.83	0.88	0.80	0.50
VE	0.80	0.71	0.84	0.81	0.55	0.68
Vt	0.67	0.64	0.73	0.83	0.56	0.64
tI	0.63	0.63	0.84	0.86	0.85	0.40
tE	0.67	0.73	0.72	0.77	0.80	0.56
tTot	0.64	0.70	0.81	0.82	0.83	0.54
tI/tE	0.45	0.32 ^#^	0.26 ^#^	0.85	0.23	0.18 ^#^
tI/tTot	0.63	0.43 ^#^	0.48	0.85	0.72	0.18 ^#^
tPTEF	0.73	0.69	0.62	0.60	0.64	0.39 ^#^
tPTIF	0.60	0.55	0.85	0.91	0.46	0.41
Compartmental Contribution Parameters						
RCpCT	0.89	0.89	0.90	0.92	0.89	0.98
RCaCT	0.82	0.83	0.85	0.84	0.85	0.99
RcCT	0.87	0.87	0.89	0.90	0.85	0.97
AbCT	0.87	0.87	0.89	0.90	0.85	0.97
RCaAbCT	0.89	0.89	0.90	0.92	0.89	0.98
RcAbIndex	0.87	0.85	0.88	0.91	0.85	0.98
Phase Angle Parameters						
RcAbPhase	0.59	0.54	0.72	0.67	0.47	0.45
RCpAbPhase	0.66	0.59	0.75	0.67	0.42 ^#^	0.52
RCpRCaPhase	0.58	0.67	0.63	0.56	0.58	0.66
RCaSPhase	0.74	0.59	0.72	0.54	0.57	0.67
RCpSPhase	0.69	0.41	0.75	0.60	0.61	0.73
AbSPhase	0.70	0.59	0.78	0.64	0.48	0.42 ^#^

^#^ ICC is not significant at the 5% level (*p* > 0.05).

## Data Availability

The data presented in this study are available on request from the corresponding author.

## References

[1] Aliverti A., Pedotti A. (2003). Opto-Electronic Plethysmography. Monaldi Arch. Chest Dis.—Pulm. Ser..

[2] Massaroni C., Carraro E., Vianello A., Miccinilli S., Morrone M., Levai I.K., Schena E., Saccomandi P., Sterzi S., Dickinson J.W. (2017). Optoelectronic Plethysmography in Clinical Practice and Research: A Review. Respiration.

[3] Massaroni C., Cassetta E., Silvestri S. (2017). A Novel Method to Compute Breathing Volumes via Motion Capture Systems: Design and Experimental Trials. J. Appl. Biomech..

[4] Smyth C.M.E., Winter S.L., Dickinson J.W. Optoelectronic Plethysmography Derived Breathing Parameters Can Differ between Athletes with and without a Dysfunctional Breathing Pattern during Exercise. Proceedings of the 2020 IEEE International Workshop on Metrology for Industry 4.0 & IoT.

[5] Ju F., Wang Y., Yin B., Zhao M., Zhang Y., Gong Y., Jiao C. (2023). Microfluidic Wearable Devices for Sports Applications. Micromachines.

[6] Zheng X., Liu Z., Liu J., Hu C., Du Y., Li J., Pan Z., Ding K. (2025). Advancing Sports Cardiology: Integrating Artificial Intelligence with Wearable Devices for Cardiovascular Health Management. ACS Appl. Mater. Interfaces.

[7] Boulding R., Stacey R., Niven R., Fowler S.J. (2016). Dysfunctional Breathing: A Review of the Literature and Proposal for Classification. Eur. Respir. Rev..

[8] Smyth C.M.E., Winter S.L., Dickinson J.W. (2022). Breathing Pattern Disorders Distinguished from Healthy Breathing Patterns Using Optoelectronic Plethysmography. Transl. Sports Med..

[9] Monaco V., Stefanini C. (2021). Assessing the Tidal Volume through Wearables: A Scoping Review. Sensors.

[10] Laufer B., Krueger-Ziolek S., Docherty P.D., Hoeflinger F., Reindl L., Moeller K. (2018). Minimum Number of Sensors in a Smart Shirt to Measure Tidal Volumes. IFAC-PapersOnLine.

[11] Laufer B., Murray R., Docherty P.D., Krueger-Ziolek S., Hoeflinger F., Reindl L., Moeller K. A Minimal Set of Sensors in a Smart-Shirt to Obtain Respiratory Parameters. Proceedings of the 21st IFAC World Congress.

[12] Massaroni C., Venanzi C., Silvatti A.P., Lo Presti D., Saccomandi P., Formica D., Giurazza F., Caponero M.A., Schena E. (2018). Smart Textile for Respiratory Monitoring and Thoraco-Abdominal Motion Pattern Evaluation. J. Biophotonics.

[13] Desiderio X., Porras C., Lunardi A.C., Marques Da Silva C.C.B., Paisani D.M., Stelmach R., Moriya H.T., Carvalho C.R.F. (2017). Comparison between the Phase Angle and Phase Shift Parameters to Assess Thoracoabdominal Asynchrony in COPD Patients. J. Appl. Physiol..

[14] Muyor J.M. (2013). Exercise Intensity and Validity of the Ratings of Perceived Exertion (Borg and OMNI Scales) in an Indoor Cycling Session. J. Hum. Kinet..

[15] Deuster P.A., Heled Y. (2008). The Sports Medicine Resource Manual.

[16] Burnley M., Jones A.M. (2007). Oxygen uptake kinetics as a determinant of sports performance. Eur. J. Sport Sci..

[17] Giavarina D. (2015). Understanding Bland Altman Analysis. Biochem. Med..

[18] Koo T.K., Li M.Y. (2016). A Guideline of Selecting and Reporting Intraclass Correlation Coefficients for Reliability Research. J. Chiropr. Med..

[19] Sureiman O., Mangera C. (2020). F-Test of Overall Significance in Regression Analysis Simplified. J. Pract. Cardiovasc. Sci..

[20] Portney L.G., Watkins M.P. (2015). Foundations of Clinical Research Applications to Practice.

[21] Vieira D.S.R., Hoffman M., Pereira D.A.G., Britto R.R., Parreira V.Ô.F. (2013). Optoelectronic Plethysmography: Intra-Rater and Inter-Rater Reliability in Healthy Subjects. Respir. Physiol. Neurobiol..

